# Effects of vincristine on the properties of low threshold mechanoreceptors and high threshold mechanoreceptors in the hindpaw glabrous skin of mice

**DOI:** 10.1186/s13041-025-01223-9

**Published:** 2025-06-17

**Authors:** Akihiro Yamada, Ayaka I. Yamada, Jennifer Ling, Jianguo G. Gu

**Affiliations:** Department of Anesthesiology and Perioperative Medicine, University of Alabama at Birmingham, Birmingham, AL 35294 United States of America

**Keywords:** Vincristine, Chemotherapy, Touch, Numbness, Neuropathic pain, Mechanical allodynia, Low threshold mechanoreceptors, High threshold mechanoreceptors

## Abstract

Vincristine is an important chemotherapy drug to treat various types of cancer, but it induces peripheral neuropathy, leading to numbness and mechanical allodynia in the hands and feet of patients. The peripheral neuropathy is a dose-limiting toxicity of vincristine chemotherapy. How vincristine treatment causes numbness and mechanical allodynia remains incompletely understood. In the present study, we utilized Nav1.8-ChR2 transgenic mice in which Nav1.8-ChR2-positive and Nav1.8-ChR2-negative mechanoreceptors could be characterized using the opto-electrophysiological method. Nav1.8-ChR2-negative Aβ- and Aδ-fiber mechanoreceptors are primarily low-threshold mechanoreceptors (LTMRs). On the other hand, Nav1.8-ChR2-positive Aβ- and Aδ-fiber mechanoreceptors are mainly high-threshold mechanoreceptors (HTMRs). We have shown that the mechanical threshold of Nav1.8-ChR2-negative Aβ-fiber mechanoreceptors, but not Nav1.8-ChR2-negative Aδ-fiber mechanoreceptors, were increased significantly in the animals treated with vincristine. In contrast, the mechanical threshold of Nav1.8-ChR2-positive Aβ-fiber mechanoreceptors were significantly reduced following vincristine treatment. Vincristine treatment did not significantly affect the mechanical sensitivity of Nav1.8-ChR2-positive Aδ- and C-fiber mechanoreceptors. Vincristine treatment also did not affect the opto-sensitivity of Nav1.8-ChR2-positive Aβ-, Aδ-, and C-fiber mechanoreceptors. Our findings suggest that mechanical sensitivity is decreased in Aβ-fiber LTMRs and increased in Aβ-HTMRs following vincristine treatment, providing insights into vincristine-induced numbness and mechanical allodynia.

## Introduction

Vincristine belongs to the category of vinca alkaloids, a class of drugs that impede the division of cancer cells [1]. Vincristine inhibits microtubule formation, thereby disrupting cell division and leading to cell death [2]. Clinically, vincristine is commonly used to treat acute lymphoblastic leukemia, Hodgkin’s and non-Hodgkin’s lymphoma, and neuroblastoma [3, 4, 5]. While effective in treating these cancers, vincristine can cause several side effects, including peripheral neuropathy in the sensory, motor, and autonomic nervous systems [6]. The vincristine-induced peripheral neuropathy in sensory nerves is characterized by numbness, tingling, and neuropathic pain in the hands and feet [6]. Vincristine-induced neuropathic pain is manifested by mechanical allodynia, excruciating pain induced by a gentle touch to the skin. These sensory dysfunctions are important clinical problems, which are the main dose-limiting toxicity of chemotherapy with vincristine [7]. Previous studies have suggested that vincristine-induced peripheral neuropathy and sensory dysfunctions are associated with its multifaceted effects on the somatosensory nervous system. The effects of vincristine on somatosensory nerves include axon demyelination and degeneration, mitochondrial dysfunction and oxidative stress, neuroinflammation and glia activation, ion channel dysregulation and neuronal hyperexcitability, alteration of sensory transmission, and activation of the immune system [8–13]. Although numbness and mechanical allodynia are the prominent symptoms of vincristine-induced peripheral neuropathy [6], it remains elusive how vincristine treatment results in these mechanosensory abnormalities.

Vincristine-induced mechanosensory abnormalities may be due to its effects on mechanoreceptors in the skin. Cutaneous mechanoreceptors are classified into several subtypes based on afferent fiber conduction velocities (Aβ-, Aδ-, and C-fibers), mechanical thresholds (low threshold mechanoreceptors, LTMRs; high threshold mechanoreceptors, HTMRs), and impulse adaptation types (slowly adapting, SA; rapidly adapting, RA) to sustained mechanical indentation [14]. LTMRs transduce tactile stimuli at the terminals of non-nociceptive afferent fibers. In the glabrous skin of mice, LTMRs are mainly Aβ-fiber RA-type LTMRs, which are the termini of Meissner’s corpuscles, and Aβ-fiber SA-type LTMRs, which comprise the Merkel cell-neurite complex [14–16]. In contrast, HTMRs are usually free nerve endings of nociceptive C- and Aδ-fibers that transduce noxious mechanical stimuli [17–20]. Although Aβ-fibers are widely believed to be LTMRs, it has been shown that a subpopulation of Aβ-fibers are HTMRs, serving as nociceptors for detecting mechanical tissue damage [20]. The properties of the LTMRs and HTMRs may be altered under pathological conditions, such as vincristine-induced peripheral neuropathy, leading to somatosensory dysfunction. However, it is currently unclear which types of cutaneous mechanoreceptors are affected by vincristine, accounting for the mechanosensory abnormalities in vincristine-induced neuropathy.

Genetics tools that specifically label distinct mechanoreceptor populations are useful for studying types of mechanoreceptors that are affected by vincristine treatment. Nav1.8-Cre mice are a transgenic mouse line that expresses Cre recombinase under the control of the promoter of Nav1.8, a sensory neuron-specific voltage-gated Na^+^ channel primarily expressed in nociceptors [22–24]. Nav1.8-Cre mouse line could be used to genetically tag nociceptive C-, Aδ-, and Aβ-fibers [24]. For example, by crossing Nav1.8-Cre mice with Ai32 (RCL-ChR2(H134R)/EYFP) mice, we have generated mice that express channel rhodopsin 2 (ChR2) under the control of Nav1.8-Cre (Nav1.8-ChR2 mice). These transgenic mice enable opto-electrophysiological studies on the properties of various mechanoreceptors [25, 26]. Utilizing Nav1.8-ChR2 mice, we have investigated the properties of mechanoreceptors in Nav1.8-ChR2-positive and Nav1.8-ChR2-negative afferent fibers innervating the hindpaws of these mice [25]. We have found that Nav1.8-ChR2-positive mechanoreceptors are mainly HTMRs, including C-fiber HTMRs, Aδ-fiber HTMRs, and Aβ-fiber HTMRs [25]. We have further shown that Nav1.8-ChR2-negative Aβ- and Aδ-fiber mechanoreceptors are primarily LTMRs [25]. Thus, Nav1.8-ChR2 mice provide a valuable transgenic model for investigating the properties of various mechanoreceptor types. Utilizing these transgenic mice, we have recently shown that Nav1.8-ChR2-positive Aβ-fiber mechanoreceptors become mechanically sensitized in tissue inflammation [26], suggesting that Aβ-HTMRs may play a role in inflammation-induced mechanical allodynia. In the present study, we aimed to investigate the effects of vincristine treatment on the properties of different mechanoreceptors in the hindpaw glabrous skin of mice.

## Materials and methods

### Animals

Nav1.8-ChR2 mice were generated by crossing Nav1.8-Cre mice with Ai32 (RCL-ChR2(H134R)/EYFP) mice. Nav1.8-Cre mice were gifts from Dr. John Wood at University College London and transferred to us from Dr. Stephen Waxman’s lab at Yale University. Ai32 mice were purchased from Jackson Labs. We crossed Nav1.8-Cre mice with Ai32 (RCL-ChR2(H134R)/EYFP) mice to generate Nav1.8^cre+^; ChR2-EYFP^loxP/+^ mouse line, hereafter termed Nav1.8-ChR2. Animal care and use conformed to NIH guidelines for care and use of experimental animals. Experimental protocols were approved by the Institutional Animal Care and Use Committee (IACUC) at the University of Alabama at Birmingham. Animals were either *i.p.* injected with saline (vehicle control group) or vincristine (vincristine treatment group). For vincristine injection, vincristine was dissolved in saline and *i.p.* injected into mice at 0.3 mg/kg daily for five consecutive days, followed by 2 days without injection, and then five consecutive days of injections.

### Ex vivo skin-nerve preparations

Nav1.8-ChR2 mice of both males and females were used at the age of 8 to 11 weeks. Skin-nerve preparations were made from the glabrous skin of the hindpaw and tibial nerves in a manner described in our previous studies [25, 27]. In brief, the animals were anesthetized with 5% isoflurane and then sacrificed by decapitation. Glabrous skin of the hindpaw, including plantar and finger regions, together with the medial plantar nerve and tibial nerve before the branch of the sciatic nerves was dissected out. The skin-nerve preparation was then placed in a 60 mm recording chamber that was coated with Sylgard Silicone on the bottom of the chamber. Fat, muscle and connective tissues on the nerves and skin were carefully removed with a pair of forceps. The skin, with the epidermis side facing up, was affixed to the bottom of the chamber by tissue pins. The nerve bundle was affixed by a tissue anchor in the same recording chamber. The cutting end of the nerve bundle was briefly exposed to a mixture of 0.05% dispase II plus 0.05% collagenase for 30–60 s, and the enzyme was then washed off by the normal Krebs solution (see below). This gentle enzyme treatment was used to help separate individual afferent fibers at the cutting end of the nerve bundle so that a single fiber could be aspirated into the recording electrode and pressure-clamped for single-fiber recordings. The recording chamber was then mounted on the stage of the Olympus BX51WI upright microscope. The skin-nerve preparation was perfused with a normal Krebs bath solution that contained (in mM): 117 NaCl, 3.5 KCl, 2.5 CaCl_2_, 1.2 MgCl_2_, 1.2 NaH_2_PO_4_, 25 NaHCO_3_, and 11 glucose (pH 7.3 and osmolarity 325 mOsm) and was saturated with 95% O_2_ and 5% CO_2_. The Krebs bath solution in the recording chamber was maintained at 28 to 32^o^C during experiments.

### Pressure-clamped single-fiber recordings

The pressure-clamped single-fiber recording was performed in a manner similar to that described in our previous studies [28] to record impulses evoked by blue LED light, mechanical indentation, and electrical stimulation. Briefly, the recording electrodes for pressure-clamped single-fiber recordings were made with thin-walled borosilicate glass tubing without filament (inner diameter 1.12 mm, outer diameter 1.5 mm, World Precision Instruments, Sarasota, FL). They were fabricated by using P-97 Flaming/Brown Micropipette Puller (Sutter Instrument Co., Novato, CA), and the tip of each electrode was fire polished with a microforge (MF-900, Narishige) to a final size of 4 to 10 μm in diameter. The recording electrode was filled with Krebs bath solution, mounted onto an electrode holder which was connected to a high-speed pressure-clamp (HSPC) device (ALA Scientific Instruments, Farmingdale, NY) for fine controls of intra-electrode pressures. Under a 40x objective, the end of the individual afferent nerve was visualized and separated by applying a low positive pressure (∼ 10 mmHg or 0.19 Psi) to the recording electrode. The end of a single nerve fiber was then aspirated into the recording electrode under a negative pressure of approximately 10 mmHg. Once the end of the nerve fiber entered the recording electrode in approximately 10 μm, the electrode pressure was readjusted to -3 ± 2 mmHg and maintained at the same pressure throughout the experiment. Nerve impulses in the single afferent fiber were recorded under the I_0_ configuration and amplified using a Multiclamp 700B amplifier (Molecular Devices, Sunnyvale, CA). Electrical signals were amplified 500 times and sampled at 25 kHz with an AC filter at 0.1 Hz and a Bessel filter at 3 kHz under an AC membrane mode (Digidata 1550B, Molecular Devices). All experiments were carried out at 30 ± 2^o^C.

To determine the conduction velocity of the recorded afferent fibers, a bipolar stimulation electrode was placed in the tibial nerve bundle and electrical stimulation was applied to the nerve bundle to evoke AP impulses. The distance between the electrical stimulation and recording sites was approximately 10 to 14 mm. Electrical stimuli were monophasic square pulses that were generated by an electronic stimulator (Master-9, A.M.P.I, Israel) with a stimulation isolator (ISO-Flex, A.M.P.I, Israel) and delivered to the stimulation electrode. The duration of each stimulation pulse was 200 µs for A-fibers and 2 ms for C-fibers, and the stimulation intensities for evoking impulses were 0.3–2.0 mA for A-fiber and 0.65–3.0 mA for C-fibers.

### Mechanical and light stimulation

For an afferent fiber being recorded, its mechanosensitive receptive field in the hindpaw glabrous skin was first searched using a glass rod. Poking the glass rod at the mechanosensitive receptive field of the recorded afferent fiber would result in the APs that were detected by the recording electrode. In the present study, all data were collected from mechanosensitive receptive sites, i.e., mechanoreceptors in the glabrous skin of the hindpaw. Once a mechanoreceptor was identified, mechanical stimulation was applied to the same receptive field by von Frey filaments to determine von Frey mechanical thresholds. The minimal force at which AP impulses were elicited by a von Frey filament was defined as the von Frey mechanical threshold for the mechanoreceptors. Mechanical stimulation was also applied to the same receptive field with a force-calibrated mechanical indenter (300 C-I, Aurora Scientific) to determine mechanical responses elicited by stepwise increases of stimulation forces. The tip size of the indenter was 0.8 mm in diameter. The indenter was connected to a Digidata 1550B Digitizer to generate ramp-and-hold mechanical stimulation using the pClamp 11 software. Before applying mechanical stimulation, the tip of the indenter was lowered to the surface of the receptive field with a 10-mN force. Then the 10-mN force was canceled to 0 so that the tip of the indenter was just in contact with the surface of the receptive field, but without having any force applied to the receptive field. Under the force control module, ramp-and-hold mechanical stimuli were applied to the mechanoreceptor of the glabrous skin. The step force commanders were calibrated by applying an indenter at finger tips, paw pads, and other areas of plantar skin, and the actual forces applied to the skin area were measured and used. The ramp-and-hold force steps were at 0, 5, 30, and 80 mN. The ramp duration (dynamic phase) was 10 ms, and the holding (static phase) was 0.98 s.

To determine whether a mechanoreceptor was from Nav1.8-ChR2-positive or Nav1.8-ChR2-negative afferent fibers, the same mechanosensitive receptive field was stimulated by a blue LED light (Thorlab; M455L4, 455 nm) to test opto-sensitivity. A mechanoreceptor was from Nav1.8-ChR2-positive afferent fibers if light stimulation evoked impulses. Otherwise, the mechanoreceptor was from opto-insensitive or Nav1.8-ChR2-negative afferent fibers. Blue LED light was applied through a 40x objective to a mechanoreceptor with a 1-s pulse of light stimulation at an intensity of 1, 10, 50 mW. Afferent impulses evoked by mechanical and light stimulation were recorded using the pressure-clamped single-fiber recordings described above.

### Data analysis

Electrophysiological data were analyzed using Clampfit 11 (Molecular Devices, Sunnyvale, CA, USA). Data were collected from 22 male and 28 female animals and aggregated for data analysis. To confirm that impulses evoked by blue light and mechanical stimulators (von Frey filaments or indenter) are generated from the same receptive field, the amplitudes and shapes of the impulses evoked by both blue LED light and mechanical stimulators were compared to ensure that the mechanically evoked impulses matched the light-evoked impulses. The conduction velocity (CV) was calculated as the distance between the stimulation site and the recording site divided by the latency of the AP impulse elicited by the electrical stimulation. All data analyses were performed using Graph Pad Prism (version 8). Unless otherwise indicated, all data were reported as individual observations and/or mean ± SEM of *n* independent observations. Statistical significance was evaluated using Mann-Whitney test, or two-way ANOVA with post hoc Bonferroni test. Differences were considered significant with *p < 0.05, **p < 0.01, ***p < 0.001, and not significant (ns) with *p* ≥ 0.05.

## Results

### Effects of vincristine treatment on the properties of Nav1.8-ChR2-negative afferent fiber mechanoreceptors in the hindpaw glabrous skin

**Fig. 1:**
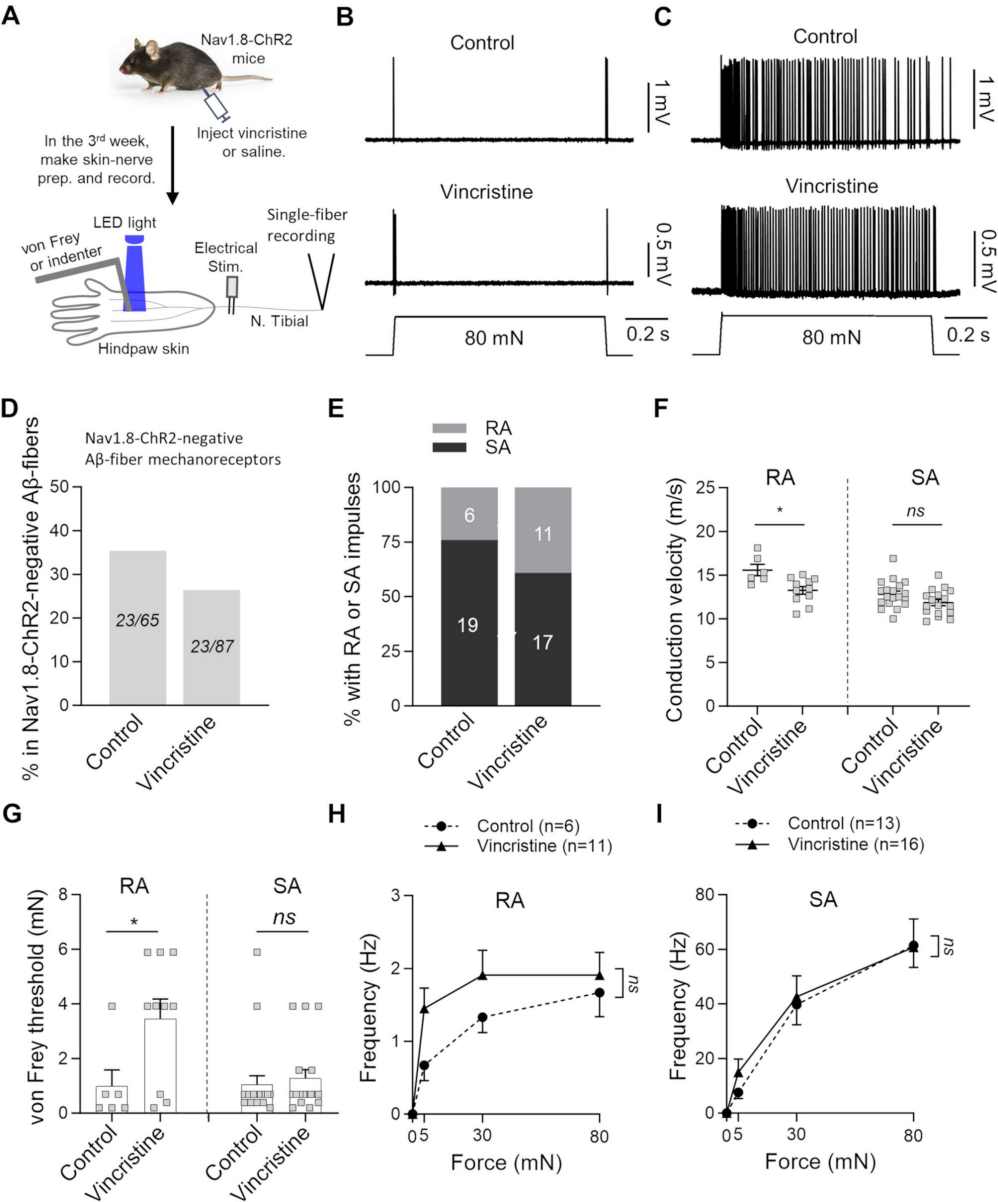
Effects of vincristine treatment on the properties of Nav1.8-ChR2-negative Aβ-fiber mechanoreceptors. (**A**) Experiment settings: Nav1.8-ChR2 mice were injected with saline (control) or vincristine. The skin-never preparation was made from the hindpaw and the tibial nerve. The pressure-clamped single-fiber recordings were conducted to measure afferent fiber conduction velocity, opto-sensitivity, and mechanical responses. (**B&C**) Representative traces illustrate RA-type (**B**) and SA-type (**C**) impulses elicited by 80-mN indentation force in Nav1.8-ChR2-negative Aβ-fiber mechanoreceptors. Top panels, control; bottom panels, vincristine-treated group. (**D**) Percent of Nav1.8-ChR2-negative Aβ-fiber mechanoreceptors in total Nav1.8-ChR2-negative Aβ-fibers. Left bar, control; right bar, vincristine-treated group. The two numbers in each bar are the Nav1.8-ChR2-negative Aβ-fiber mechanoreceptors and the total Nav1.8-ChR2-negative Aβ-fibers recorded. (**E**) Percent of Nav1.8-ChR2-negative Aβ-fiber mechanoreceptors being RA-type or SA-type in the control and vincristine-treated group. The two numbers in each bar are the RA-type and SA-type being recorded. (**F**) Conduction velocity of RA-type (left panel) and SA-type (right panel) Nav1.8-ChR2-negative Aβ-fiber mechanoreceptors in the control and vincristine-treated group. (**G**) von Frey thresholds for eliciting impulses by von Frey filaments in Nav1.8-ChR2-negative Aβ-fiber mechanoreceptors in the control and vincristine-treated group. Left panel, RA-type; right panel, SA-type. (**H**) Impulse frequency induced by different indentation forces in RA-type Nav1.8-ChR2-negative Aβ-fiber mechanoreceptors in the control (*n* = 6) and vincristine treated group (*n* = 11). (**I**) Impulse frequency induced by different indentation forces in SA-type Nav1.8-ChR2-negative Aβ-fiber mechanoreceptors in the control (*n* = 13) and vincristine treated group (*n* = 16). Indentation forces were delivered to the receptive field via a calibrated indenter in both (**H** and (**I**. Data present individual observations and/or mean ± SEM, *p < 0.05, **p < 0.01, ns, no significant difference, Mann-Whitney test or two-way ANOVA followed by Bonferroni’s post hoc test

In the present study, we made the skin-nerve preparations from saline-injected (control) and vincristine-injected (vincristine-treated) Nav1.8-ChR2 mice. We employed pressure-clamped single-fiber recordings to investigate the properties of mechanoreceptors in the hindpaw glabrous skin in the control and vincristine treated group (Fig. 1A). These properties of the afferent fiber mechanoreceptors include opto-sensitivity, mechanical sensitivity, and conduction velocity. The afferent fiber mechanoreceptors were classified as Nav1.8-ChR2-negative (opto-insensitive) and Nav1.8-ChR2-positive (opto-sensitive) fibers based on their sensitivity to blue LED light stimulation. Furthermore, we classified the mechanoreceptors into Aβ-fiber (CV ≥ 9 m/s), Aδ-fiber (9 m/s > CV > 1.2 m/s), and C-fiber (CV ≤ 1.2 m/s) based on their conduction velocities [20, 25, 29].

Figure 1 shows our investigation on the effects of vincristine treatment on Nav1.8-ChR2-negative Aβ-fiber mechanoreceptors. Nav1.8-ChR2-negative Aβ-fiber mechanoreceptors in both control and vincristine-treated groups showed rapidly adapting (RA-type, Fig. 1B) or slowly adapting (SA-type, Fig. 1C) impulses in response to stepwise mechanical stimulation. The Nav1.8-ChR2-negative Aβ-fiber mechanoreceptors account for 35% (23/65) of the total Nav1.8-ChR2-negative Aβ- fibers in the control group and 26% (23/87) of the total Nav1.8-ChR2-negative Aβ-fibers in the vincristine-treated group (Fig. 1D). Of all Nav1.8-ChR2-negative Aβ-fiber mechanoreceptors, 24% of them were RA-type and 76% were SA-type in the control group; 39% of them were RA-type and 61% were SA-type in vincristine-treated group (Fig. 1E). There was no significant difference in the proportion of RA-type and SA-type between the control and the vincristine-treated groups. Vincristine treatment led to a significant reduction in the conduction velocity (CV) of the RA-type Nav1.8-ChR2-negative Aβ-fiber mechanoreceptors, with a CV of 15.6 ± 0.6 m/s (*n* = 6) in the control group and reduced to 13.3 ± 0.4 m/s (*n* = 11, *p* < 0.01) in the vincristine-treated group (Fig. 1F left panel). For the SA-type Nav1.8-ChR2-negative Aβ-fiber mechanoreceptors, the CV was 12.8 ± 0.4 m/s (*n* = 19) in the control group and 11.0 ± 0.3 m/s (*n* = 17) in the vincristine-treated groups, and there was no significant difference between them (Fig. 1F right panel). We examined whether vincristine treatment affects mechanical sensitivity in Nav1.8-ChR2-negative Aβ-fiber mechanoreceptors. Vincristine treatment significantly increased the von Frey threshold in the RA-type Nav1.8-ChR2-negative Aβ-fiber mechanoreceptors, from 0.4 ± 0.1 mN (*n* = 6) in the control group to 3.5 ± 0.7 mN (*n* = 10, *p* < 0.05) in the vincristine-treated group (Fig. 1G, left panel). However, there was no significant difference in the von Frey threshold of the SA-type Nav1.8-ChR2-negative Aβ-fiber mechanoreceptors between the control and vincristine-treated group (Fig. 1G, right panel). Using a force-calibrated mechanical indenter, we determined mechanical responses elicited by stepwise indentation forces of 5, 30, and 80 mM (Fig. 1H&I). Impulse frequency was increased with increased indentation forces in the RA-type Nav1.8-ChR2-negative Aβ-fiber mechanoreceptors in the control (*n* = 6) and vincristine-treated groups (*n* = 11) (Fig. 1H). Similarly, impulse frequency was increased with enhanced indentation forces in the SA-type Nav1.8-ChR2-negative Aβ-fiber mechanoreceptors in the control (*n* = 13) and vincristine-treated groups (*n* = 16) (Fig. 1I). However, there was no significant difference in the mechanical responses, as measured by the force-frequency relationship, between the control and vincristine-treated groups (Fig. 1H&I).

**Fig. 2:**
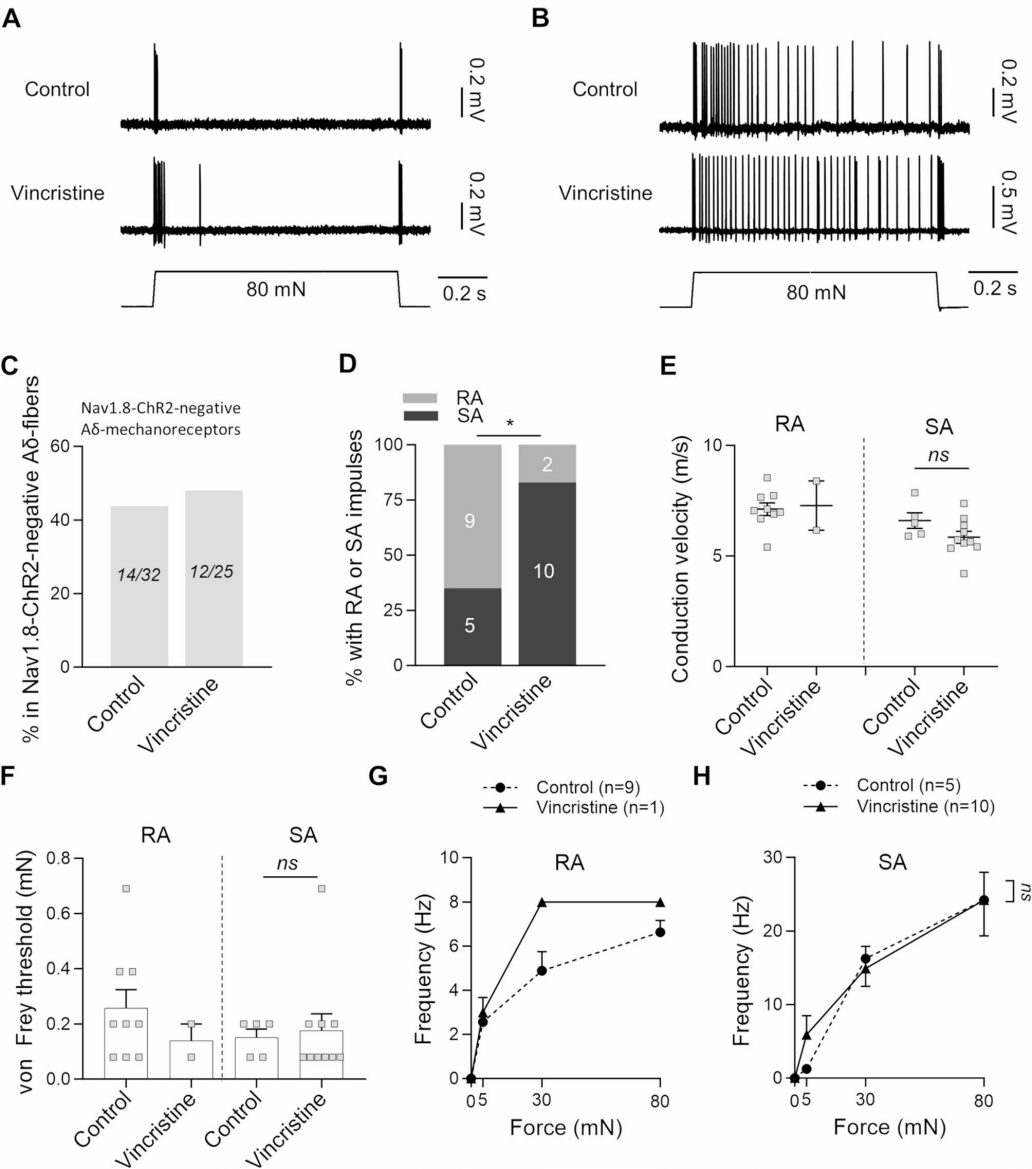
Effects of vincristine treatment on the properties of Nav1.8-ChR2-negative Aδ-fiber mechanoreceptors. (**A&B**) Representative traces illustrate RA-type (**A**) and SA-type (**B**) impulses elicited by 80-mN indentation force in Nav1.8-ChR2-negative Aδ-fiber mechanoreceptors. Top panels, control; bottom panels, vincristine-treated group. (**C**) Percent of Nav1.8-ChR2-negative Aδ-fiber mechanoreceptors in total Nav1.8-ChR2-negative Aδ-fibers. Left bar, control; right bar, vincristine-treated group. The numbers in each bar are the Nav1.8-ChR2-negative Aδ-fiber mechanoreceptors and the total Nav1.8-ChR2-negative Aδ-fibers recorded. (**D**) Percent of Nav1.8-ChR2-negative Aδ-fiber mechanoreceptors being RA-type or SA-type in the control and vincristine-treated group. The two numbers in each bar are the RA-type and SA-type being recorded. (**E**) Conduction velocity of RA-type (left panel) and SA-type (right panel) of Nav1.8-ChR2-negative Aδ-fiber mechanoreceptors in the control and vincristine-treated group. (**F**) von Frey thresholds for eliciting impulses by von Frey filaments in Nav1.8-ChR2-negative Aδ-fiber mechanoreceptors in the control and vincristine-treated group. Left panel, RA-type; right panel, SA-type. (**G**) Impulse frequency induced by different mechanical forces in RA-type Nav1.8-ChR2-negative Aδ-fiber mechanoreceptors in the control (*n* = 9) and vincristine treated group (*n* = 1). (**H**) Impulse frequency induced by different indentation forces in SA-type Nav1.8-ChR2-negative Aδ-fiber mechanoreceptors in the control (*n* = 5) and vincristine treated group (*n* = 10). Indentation forces were delivered to the receptive field via a calibrated indenter in both G and H. Data present individual observations and/or mean ± SEM, ns, no significant difference, Mann-Whitney test or two-way ANOVA followed by Bonferroni’s post hoc test

We examined the effects of vincristine on the properties of Nav1.8-ChR2-negative Aδ-fiber mechanoreceptors (Fig. 2). Nav1.8-ChR2-negative Aδ-fiber mechanoreceptors in both control and vincristine-treated groups exhibited RA-type (Fig. 2A) or SA-type (Fig. 2B) impulses in response to stepwise mechanical stimulation. Overall, the Nav1.8-ChR2-negative Aδ-fiber mechanoreceptors account for 44% (14/32) of Nav1.8-ChR2-negative Aδ-fibers in the control group, and account for 48% (12/25) of Nav1.8-ChR2-negative Aδ-fibers in the vincristine-treated group (Fig. 2C). Of all Nav1.8-ChR2-negative Aδ-fiber mechanoreceptors, 64% (9/14) of them were RA-type and 36% (5/14) were SA-type in control group; 17% (2/12) of them were RA-type and 83% (10/12) were SA-type in vincristine-treated group (Fig. 2D). Vincristine treatment significantly decreased RA-type and increased SA-type of Nav1.8-ChR2-negative Aδ-fiber mechanoreceptors (*p* < 0.05, Fig. 2D). We examined the effects of vincristine on the conduction velocity of Nav1.8-ChR2-negative Aδ-fiber mechanoreceptors. For the RA-type Nav1.8-ChR2-negative Aδ-fiber mechanoreceptors, the CV was 7.1 ± 0.3 m/s (*n* = 9) in the control group and 7.3 ± 1.1 m/s (*n* = 2) in the vincristine-treated group (Fig. 2E, left panel). Statistical comparison was not made due to the small sample size in the vincristine-treated group. For the SA-type Nav1.8-ChR2-negative Aδ-fiber mechanoreceptors, the CV was 6.6 ± 0.4 m/s (*n* = 5) in the control group and 5.9 ± 0.3 m/s (*n* = 10) in the vincristine-treated group (Fig. 2E, left panel), and there was no significant difference in the CV between the control and vincristine-treated groups. Effects of vincristine treatment on mechanical sensitivity in Nav1.8-ChR2-negative Aδ-fiber mechanoreceptors were examined. The von Frey threshold in the RA-type Nav1.8-ChR2-negative Aδ-fiber mechanoreceptors was 0.26 ± 0.07 mN (*n* = 9) in the control group and 0.14 ± 0.6 mM (*n* = 2) in the vincristine-treatment group (Fig. 2F); statistical comparison was not made due to the small sample size in the vincristine-treated group. For the SA-type Nav1.8-ChR2-negative Aδ-fiber mechanoreceptors, the von Frey threshold was 0.15 ± 0.07 mN (*n* = 5) in the control group and 0.18 ± 0.6 mM (*n* = 10) in the vincristine-treatment group, and there was no significant difference between the control and vincristine-treated group (Fig. 2F). There was also no significant difference in the mechanical responses, as measured by the force-frequency relationship, between the control and vincristine-treated groups (Fig. 2G&H).

### Effects of vincristine treatment on the properties of Nav1.8-ChR2-positive afferent fiber mechanoreceptors in the hindpaw glabrous skin

**Fig. 3:**
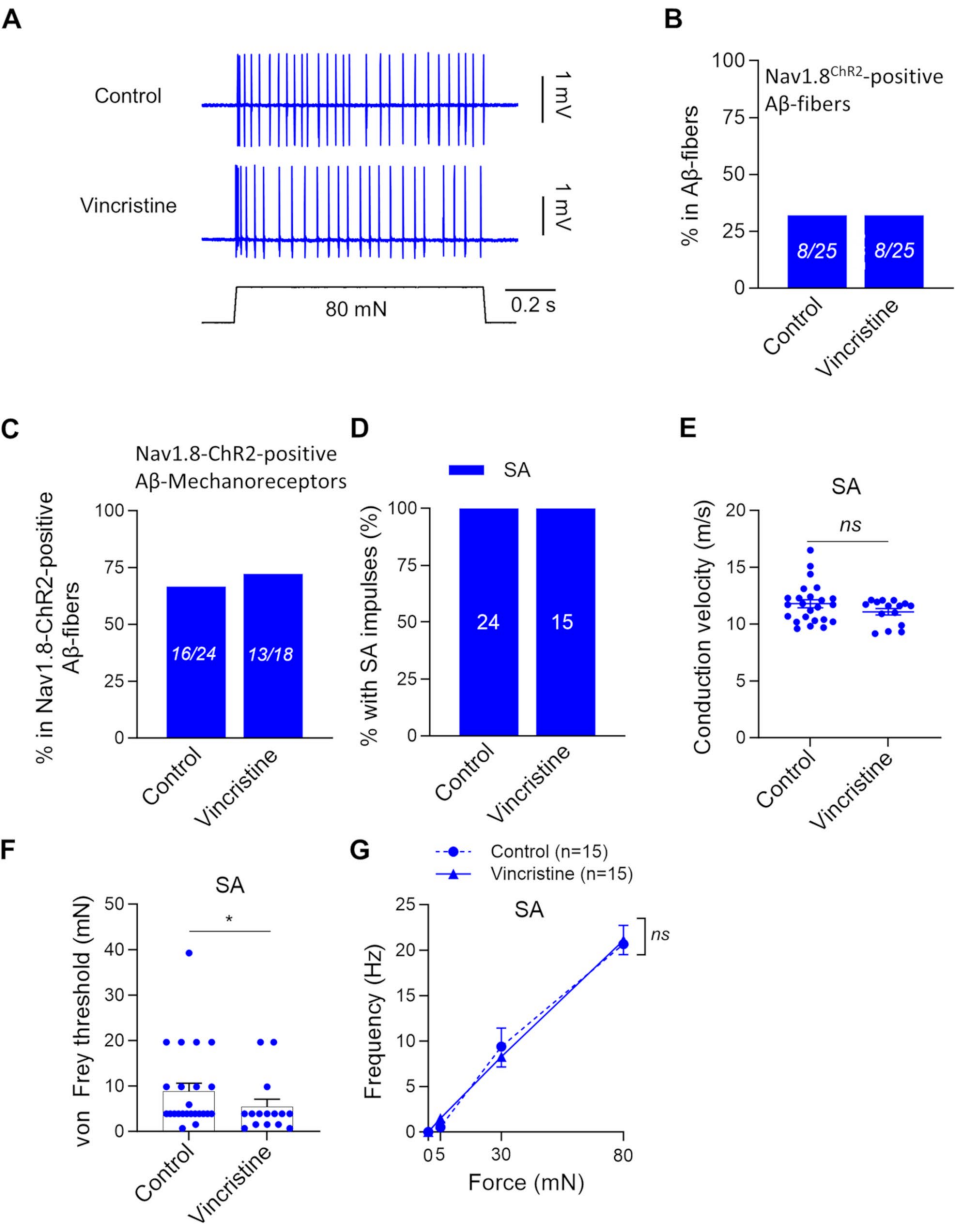
Effects of vincristine treatment on the properties of Nav1.8^−^ChR2-positive Aβ-fiber mechanoreceptors. (**A**) Representative traces illustrate SA-type impulses elicited by 80-mN indentation force in Nav1.8-ChR2-positive Aβ-fiber mechanoreceptors. Top panels, control; bottom panels, vincristine-treated group. (**B**) Percent of Nav1.8-ChR2-positive Aβ-fibers in total Aβ-fibers. Left bar, control; right bar, vincristine-treated group. The numbers in each bar are the Nav1.8-ChR2-positive Aβ-fibers and the total Aβ-fibers recorded. (**C**) Percent of Nav1.8-ChR2-positive Aβ-fiber mechanoreceptors in total Nav1.8-ChR2-positive Aβ-fibers. Left bar, control; right bar, vincristine-treated group. The numbers in each bar are the Nav1.8-ChR2-positive Aβ-fiber mechanoreceptors and the total Nav1.8-ChR2-positive Aβ-fibers recorded. (**D**) Percent of Nav1.8-ChR2-positive Aβ-fiber mechanoreceptors being SA-type in the control (*n* = 24) and vincristine-treated group (*n* = 15). (**E**) Conduction velocity of SA-type Nav1.8-ChR2-positive Aβ-fiber mechanoreceptors in the control and vincristine-treated group. (**F**) von Frey thresholds for eliciting impulses by von Frey filaments in Nav1.8-ChR2-positive Aβ-fiber mechanoreceptors in the control and vincristine-treated group. (**G**) Impulse frequency induced by different indentation forces in SA-type Nav1.8-ChR2-positive Aβ-fiber mechanoreceptors in the control (*n* = 15) and vincristine treated group (*n* = 15). Indentation forces were delivered to the receptive field via a calibrated indenter in G. Data present individual observations and/or mean ± SEM, ns, no significant difference, Mann-Whitney test or two-way ANOVA followed by Bonferroni’s post hoc test

We examined the effects of vincristine treatment on the properties of Nav1.8-ChR2-positive afferent fiber mechanoreceptors. Nav1.8-ChR2-positive afferent fiber mechanoreceptors were the afferent fiber endings in the hindpaw glabrous skin that are both opto-sensitive and mechanosensitive. In both the control and vincristine-treated groups, the Nav1.8-ChR2-positive afferent fiber mechanoreceptors with CV in the Aβ-fiber range (CV ≥ 9 m/s), i.e., Aβ-fiber mechanoreceptors, responded to stepwise mechanical stimulation with SA-type impulses (Fig. 3A). The overall proportions of Nav1.8-ChR2-positive Aβ-fibers in all Aβ-fibers were 32% (8/25) in the control group, and also were 32% (8/25) in the vincristine-treated group (Fig. 3B). Of all Nav1.8-ChR2-positive Aβ-fibers, Nav1.8-ChR2-positive Aβ-fiber mechanoreceptors account for 67% (16/24) in the control group and 72% (13/18) in the vincristine-treated group (Fig. 3C). Of all Nav1.8-ChR2-positive Aβ-fiber mechanoreceptors, all were SA-type in the control (100%, 24/24) and vincristine-treated group (100%, 15/15), and no RA-type was encountered in the control and vincristine-treated groups (Fig. 3D). The conduction velocity of Nav1.8-ChR2-positive Aβ-fiber mechanoreceptors was 11.8 ± 0.4 m/s (*n* = 24) in the control group and 11.1 ± 0.28 m/s (*n* = 15) in the vincristine-treated group, and there was no significant change after vincristine treatment. The von Frey threshold for the Nav1.8-ChR2-positive Aβ-fiber mechanoreceptors showed a significant reduction following vincristine treatment, with the control group being 8.8 ± 1.8 mN (*n* = 24) and the vincristine-treated group being 5.5 ± 1.5 mN (*n* = 15, *p* < 0.05, Fig. 3F). However, the mechanical responses of the Nav1.8-ChR2-positive Aβ-fiber mechanoreceptors, as measured by the force-frequency relationship, showed no significant difference between the control and vincristine-treated groups (Fig. 3G).

**Fig. 4:**
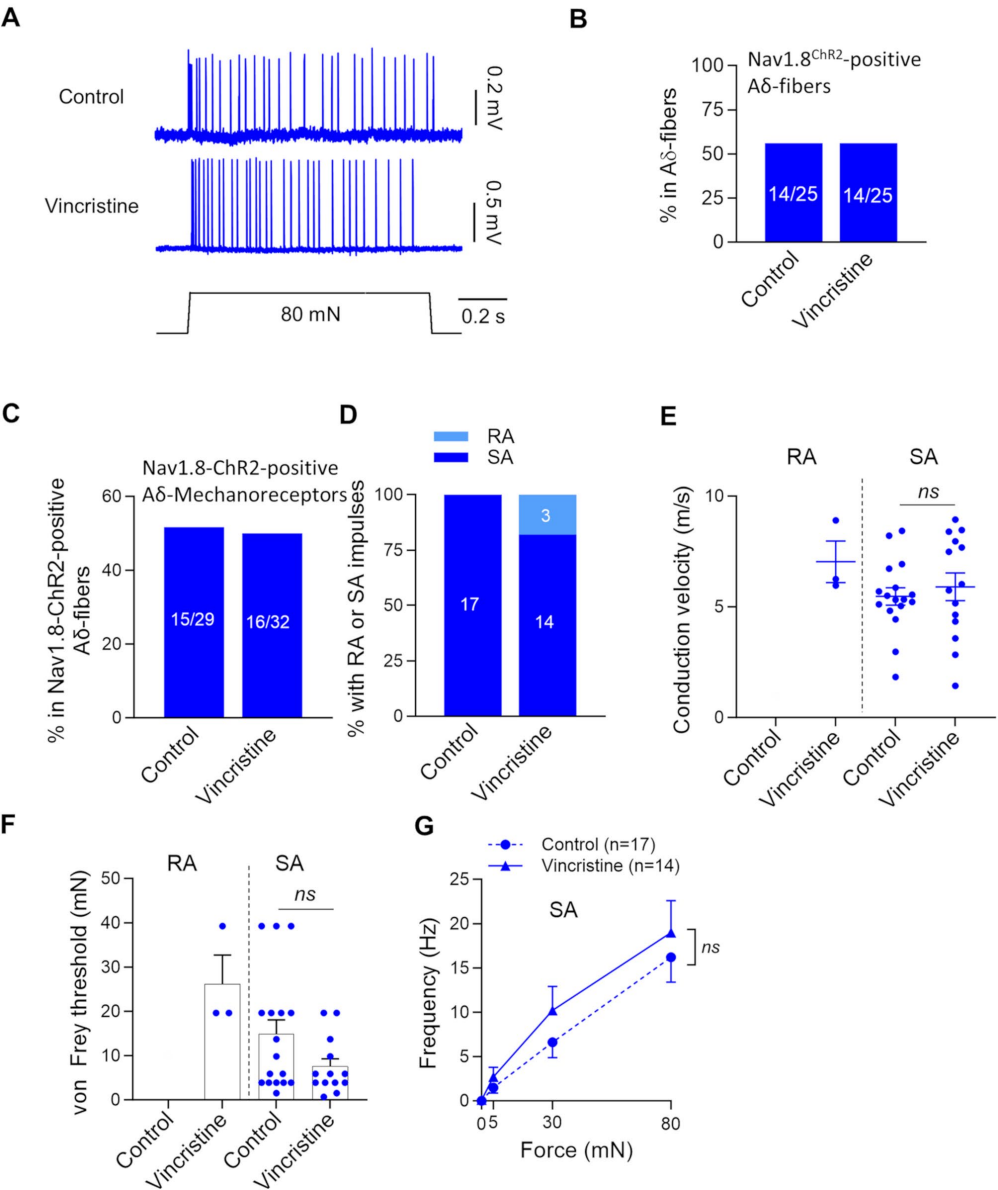
Effects of vincristine treatment on properties of Nav1.8-ChR2-positive Aδ-fiber mechanoreceptors. (**A**) Representative traces illustrate SA-type impulses elicited by 80-mN indentation force in Nav1.8-ChR2-positive Aδ-fiber mechanoreceptors. Top panels, control; bottom panels, vincristine-treated group. (**B**) Percent of Nav1.8-ChR2-positive Aδ-fibers in total Aδ-fibers. Left bar, control; right bar, vincristine-treated group. The numbers in each bar are the Nav1.8-ChR2-positive Aδ-fibers and the total Aδ-fibers recorded. (**C**) Percent of Nav1.8-ChR2-positive Aδ-fiber mechanoreceptors in total Nav1.8-ChR2-positive Aδ-fibers. Left bar, control; right bar, vincristine-treated group. The numbers in each bar are the Nav1.8-ChR2-positive Aδ-fiber mechanoreceptors and the total Nav1.8-ChR2-positive Aδ-fibers recorded. (**D**) Percent of Nav1.8-ChR2-positive Aδ-fiber mechanoreceptors being SA-type (*n* = 17) and RA (*n* = 0) in the control, and SA-type (*n* = 14) and RA (*n* = 3) in the vincristine-treated group. (**E**) Conduction velocity of Nav1.8-ChR2-positive Aδ-fiber mechanoreceptors in the control and vincristine-treated group. Left panel, RA-type; right panel, SA-type. (**F**) von Frey thresholds for eliciting impulses by von Frey filaments in Nav1.8-ChR2-positive Aδ-fiber mechanoreceptors in the control and vincristine-treated group. Left panel, RA-type; right panel, SA-type. (**G**) Impulse frequency induced by different mechanical forces in SA-type Nav1.8-ChR2-positive Aδ-fiber mechanoreceptors in the control (*n* = 17) and vincristine treated group (*n* = 14). Indentation forces were delivered to the receptive field via a calibrated indenter in G. Data present individual observations and/or mean ± SEM, ns, no significant difference, Mann-Whitney test or two-way ANOVA followed by Bonferroni’s post hoc test

Effects of vincristine treatment on Nav1.8-ChR2-positive Aδ-fiber mechanoreceptors were examined (Fig. 4). SA-type impulses could be elicited from Nav1.8-ChR2-positive Aδ-fiber mechanoreceptors following stepwise mechanical stimulation in both the control and vincristine-treated groups (Fig. 4A). Nav1.8-ChR2-positive Aδ-fiber afferents accounted for 56% (14/25) of all Aδ-fibers in both the control group and vincristine-treated group (Fig. 4B). Of Nav1.8-ChR2-positive Aδ-fibers, 52% (15/29) and 50% (16/32) were Nav1.8-ChR2-positive Aδ-fiber mechanoreceptors in the control group and the vincristine-treated group, respectively (Fig. 4C). All (17/17) Nav1.8-ChR2-positive Aδ-fiber mechanoreceptors displayed SA-type impulses and no RA-type impulses was encountered in the control group (Fig. 4D). SA-type impulses remain to be the dominant type (82%, 14/17) in Nav1.8-ChR2-positive Aδ-fiber mechanoreceptors, although RA-type appeared in 18% (3/17) of Nav1.8-ChR2-positive Aδ-fiber mechanoreceptors in the vincristine-treated group (Fig. 4D). The conduction velocity of RA-type Nav1.8-ChR2-positive Aδ-fiber mechanoreceptors was not obtained in the control group (*n* = 0), and was 7.0 ± 0.9 m/s (*n* = 3) in the vincristine-treated group (Fig. 4E, left panel). The conduction velocity of the SA-type Nav1.8-ChR2-positive Aδ-fiber mechanoreceptors was 5.4 ± 0.4 m/s (*n* = 17) in the control group, and 5.9 ± 0.6 m/s (*n* = 14) in the vincristine-treated group (Fig. 4E, right panel), and there was no significant difference between them. The von Frey threshold for the RA-type Nav1.8-ChR2-positive Aδ-fiber mechanoreceptors was not obtained in the control group (*n* = 0), and was 26.2 ± 6.5 mN (*n* = 3) in the vincristine-treated group (Fig. 4F, left panel). The SA-type Nav1.8-ChR2-positive Aδ-fiber mechanoreceptors showed no significant change in the von Frey threshold following vincristine treatment, was 14.9 ± 3.2 mN (*n* = 17) in the control group and 7.6 ± 1.8 mN in the vincristine-treated group (*n* = 13, Fig. 3F, right panel). The mechanical responses of the Nav1.8-ChR2-positive Aδ-fiber mechanoreceptors, as measured by the force-frequency relationship, also showed no significant difference between the control and vincristine-treated groups (Fig. 4G).

**Fig. 5:**
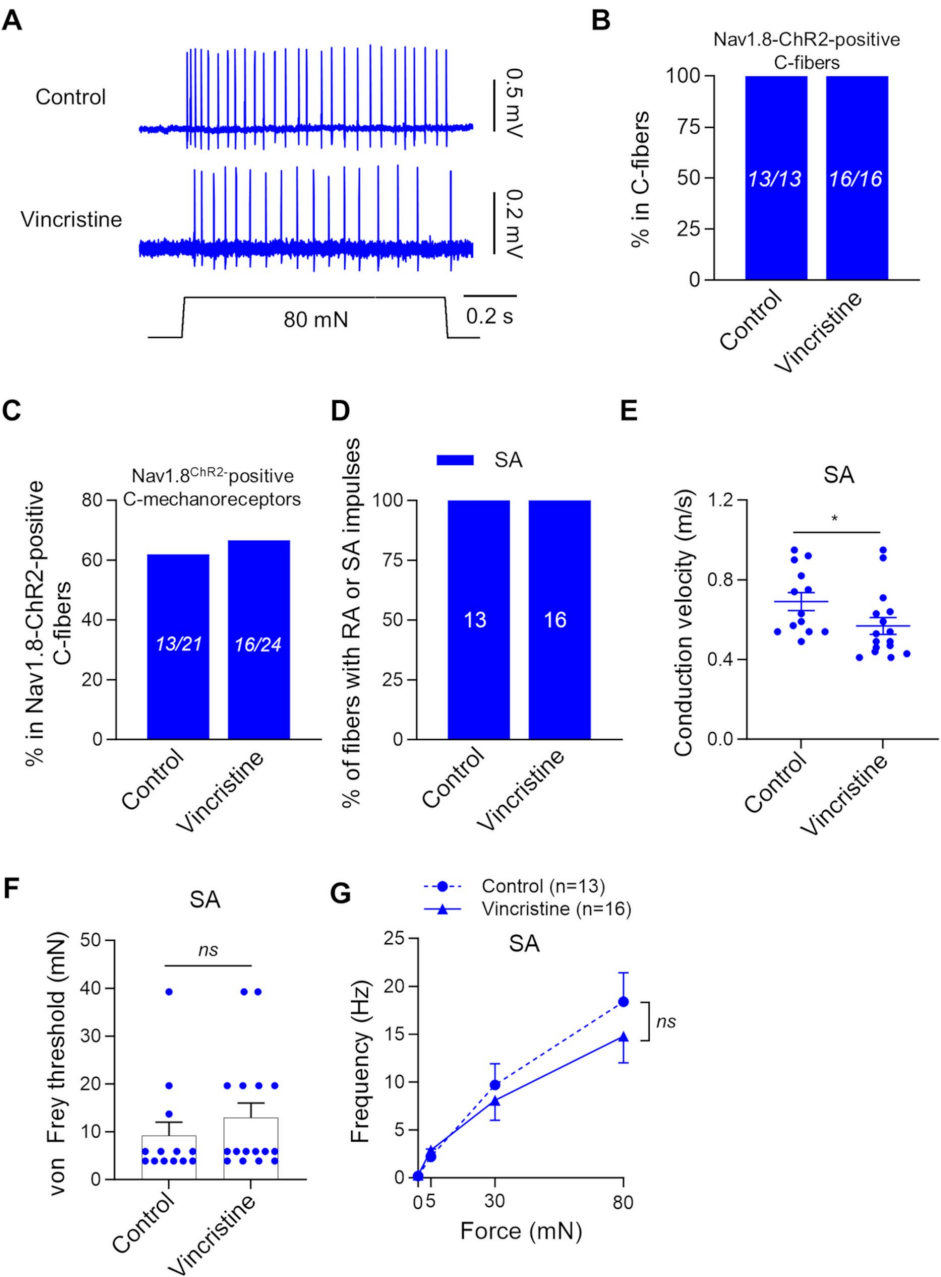
Effects of vincristine treatment on the properties of Nav1.8-ChR2-positive C-fiber mechanoreceptors. (**A**) Representative traces illustrate SA-type impulses elicited by 80-mN indentation force in Nav1.8-ChR2-positive C-fiber mechanoreceptors. Top panels, control; bottom panels, vincristine-treated group. (**B**) Percent of Nav1.8-ChR2-positive C-fibers in total C-fibers. Left bar, control; right bar, vincristine-treated group. The numbers in each bar are the Nav1.8-ChR2-positive C-fibers and the total C-fibers recorded. (**C**) Percent of Nav1.8-ChR2-positive C-fiber mechanoreceptors in total Nav1.8-ChR2-positive C-fibers. Left bar, control; right bar, vincristine-treated group. The numbers in each bar are the Nav1.8-ChR2-positive C-fiber mechanoreceptors and the total Nav1.8-ChR2-positive C-fibers recorded. (**D**) Percent of Nav1.8-ChR2-positive C-fiber mechanoreceptors being SA-type (*n* = 13) in the control, and SA-type (*n* = 16) in the vincristine-treated group. (**E**) Conduction velocity of Nav1.8-ChR2-positive C-fiber mechanoreceptors in the control and vincristine-treated group. (**F**) von Frey thresholds for eliciting impulses by von Frey filaments in Nav1.8-ChR2-positive C-fiber mechanoreceptors in the control and vincristine-treated group. (**G**) Impulse frequency induced by different mechanical forces in Nav1.8-ChR2-positive C-fiber mechanoreceptors in the control (*n* = 13) and vincristine treated group (*n* = 16). Indentation forces were delivered to the receptive field via a calibrated indenter in G. Data present individual observations and/or mean ± SEM, ns, no significant difference, Mann-Whitney test or two-way ANOVA followed by Bonferroni’s post hoc test

We examined the effects of vincristine on Nav1.8-ChR2-positive C-fiber mechanoreceptors (Fig. 5). SA-type impulses were elicited from Nav1.8-ChR2-positive C-fiber mechanoreceptors following stepwise mechanical stimulation (Fig. 5A). All C-fibers were Nav1.8-ChR2-positive in the control group (100%, 13/13) and vincristine-treated group (100%, 16/16, Fig. 5B). Of the Nav1.8-ChR2-positive C-fibers, 62% (13/21) and 67% (16/24) were Nav1.8-ChR2-positive C-fiber mechanoreceptors in the control group and the vincristine-treated group, respectively (Fig. 5C). All Nav1.8-ChR2-positive C-fiber mechanoreceptors were SA-type in the control group (100%, 13/13) and vincristine-treated group (100%, 16/16), and no RA-type was encountered in the control and vincristine-treated group (Fig. 5D). The conduction velocity of the SA-type Nav1.8-ChR2-positive C-fiber mechanoreceptors was 0.69 ± 0.04 m/s (*n* = 13) in the control group, and significantly reduced to 0.57 ± 0.04 m/s (*n* = 16. *P* < 0.05) in the vincristine-treated group (Fig. 5E). The von Frey threshold for the Nav1.8-ChR2-positive C-fiber mechanoreceptors was not significantly different between the control group (9.2 ± 2.8 mN, *n* = 13) and vincristine-treated group (13.0 ± 3.0 mN, *n* = 16, Fig. 5F). The mechanical responses of the Nav1.8-ChR2-positive C-fiber mechanoreceptors, as measured by the force-frequency relationship, also had no significant difference between the control and vincristine-treated groups (Fig. 5G).

**Fig. 6:**
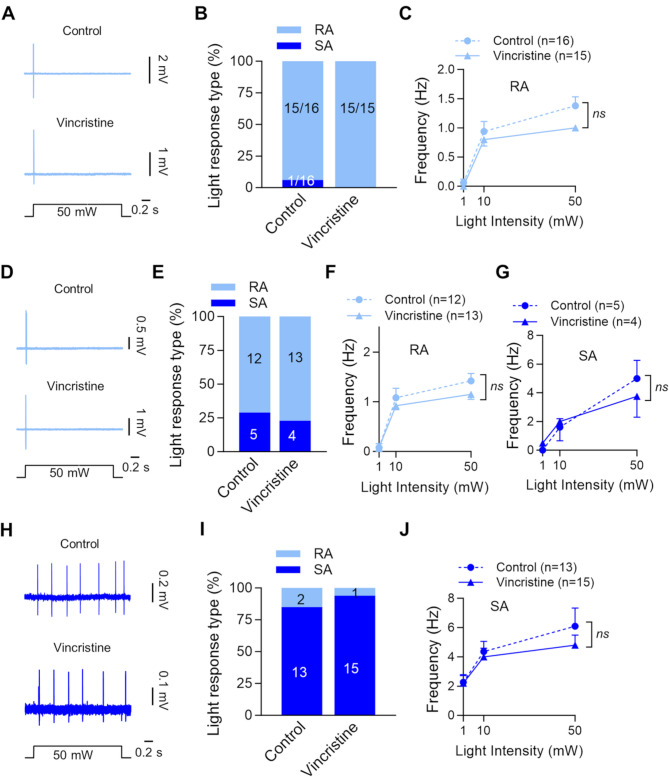
Effects of vincristine on opto-sensitivity in Nav1.8-ChR2-positive Aβ-, Aδ, and C-fiber mechanoreceptors. (**A**) Representative traces illustrate blue light-elicited impulses from Nav1.8-ChR2-positive Aβ-fiber mechanoreceptors in the control (top panel) and vincristine treated group. The mechano-sensitive receptive field was stimulated by a focused beam of 50-mW blue LED light for 1 s. (**B**) Proportions of Nav1.8-ChR2-positive Aβ-fiber mechanoreceptors with RA-type or SA-type impulses in the control (RA, *n* = 15; SA, *n* = 1), vincristine-treated group (RA, *n* = 15; SA, *n* = 0). (**C**) RA impulses elicited by blue LED light at the intensities of 1, 10, and 50 mW in the control (*n* = 16) and vincristine-treated group (*n* = 15). (**D-G**) Similar to A-C, except Nav1.8-ChR2-positive Aδ-fiber mechanoreceptors were tested. (**H-J**) Similar to A-C, except Nav1.8-ChR2-positive C-fiber mechanoreceptors were tested. Data present mean ± SEM, ns, no significant difference, two-way ANOVA followed by Bonferroni’s post hoc test

We characterized the opto-sensitivity of Nav1.8-ChR2-positive Aβ-, Aδ-, and C-fiber mechanoreceptors (Fig. 6). For Nav1.8-ChR2-positive Aβ-fiber mechanoreceptors, RA-type responses, typically a single impulse, were elicited at the beginning of the blue LED light stimulation in both the control group (15/16) and the vincristine-treated group (15/15, Fig. 6A&B). Only 1 in 16 Nav1.8-ChR2-positive Aβ-fiber mechanoreceptors displayed SA-type impulses in the control group (Fig. 6B), and no Nav1.8-ChR2-positive Aβ-fiber mechanoreceptors showed SA-type impulses in the vincristine-treated group (Fig. 6B). Figure 6C shows the frequency of the impulses elicited by blue LED light at the intensities of 1, 10, and 50 mW. There was no significant difference in the opto-sensitivity between the control and vincristine-treated groups (Fig. 6C).

Nav1.8-ChR2-positive Aδ-fiber mechanoreceptors mostly responded to blue LED light stimulation with RA-type impulses in both the control and vincristine-treated groups (Fig. 6D&E). However, a small portion of Nav1.8-ChR2-positive Aδ-fiber mechanoreceptors showed SA-type impulses sustained through the period of light stimulation in both the control group and vincristine-treated group (Fig. 6E). In the control group, of 17 Nav1.8-ChR2-positive Aδ-fiber mechanoreceptors tested, 71% (12/17) showed RA-type impulses and 29% (5/17) showed SA-type impulses. In the vincristine-treated group, of 17 Nav1.8-ChR2-positive Aδ-fiber mechanoreceptors tested, 76% (13/17) showed RA-type impulses, and 24% (4/17) displayed SA-type impulses. The response frequency of the RA-type (Fig. 6F) and SA-type impulses (Fig. 6G) at different stimulation intensities (1, 10, and 50 mW) of blue LED light showed no significant differences between the control group and vincristine-treated group (Fig. 6F&G). In contrast to Nav1.8-ChR2-positive Aβ- and Aδ-fiber mechanoreceptors, Nav1.8-ChR2-positive C-fiber mechanoreceptors typically showed SA-type impulses during blue LED light stimulation (Fig. 6H&I). In the control group, 13 in 15 Nav1.8-ChR2-positive C-fiber mechanoreceptors displayed SA-type impulses (Fig. 6I). In the vincristine-treated group, 15 in 16 Nav1.8-ChR2-positive C-fiber mechanoreceptors showed SA-type impulses (Fig. 6I). The response frequency of the SA-type impulses elicited at different stimulation intensities (1, 10, and 50 mW) of blue LED light showed no significant differences between the control group and vincristine-treated group (Fig. 6J).

## Discussion

In the present study, we investigated the effects of the chemotherapy drug vincristine on different types of mechanoreceptors in the hindpaw glabrous skin of Nav1.8-ChR2 mice. A main finding of this study is that the RA-type Nav1.8-ChR2-negative Aβ-fiber mechanoreceptors exhibited a decreased mechanical sensitivity (increased von Frey threshold) following vincristine treatment. Furthermore, SA-type Nav1.8-ChR2-positive Aβ-fiber mechanoreceptors show an increased mechanical sensitivity (reduced von Frey threshold) in vincristine-treated animals. These findings may provide some insights into somatosensory disorders, including numbness and mechanical allodynia, seen clinically in patients administrated with vincristine as a chemotherapy drug.

Nav1.8-ChR2 mice were utilized in the present study, enabling us to employ the opto-electrophysiological approach to distinguish between Nav1.8-ChR2-negative (opto-insensitive) and Nav1.8-ChR2-positive (opto-sensitive) afferent fiber mechanoreceptors, similar to our previous studies [25, 26]. We show that Nav1.8-ChR2-negative Aβ-fiber mechanoreceptors displayed either RA- or SA-types of impulses. We have found that the conduction velocity is significantly reduced in RA-type Nav1.8-ChR2-negative Aβ-fiber mechanoreceptors and marginally decreased in SA-type Nav1.8-ChR2-negative Aβ-fiber mechanoreceptors. Consistently, previous studies have also shown that vincristine treatment decreases A-fiber and C-fiber conduction velocity in rats [30]. We have demonstrated that in the control group, most Nav1.8-ChR2-negative Aβ-fiber mechanoreceptors, both RA- and SA-types, have von Frey thresholds of less than 4 mN. This finding is consistent with our previous study [25], indicating that Nav1.8-ChR2-negative Aβ-fiber mechanoreceptors are primarily low-threshold Aβ-fiber mechanoreceptors (Aβ-LTMRs). We have demonstrated that vincristine treatment significantly increases the von Frey mechanical threshold, i.e., decreases the mechanical sensitivity, of RA-type Nav1.8-negative Aβ-fiber mechanoreceptors. This result may suggest that the mechanical sensitivity of Meissner’s corpuscles in the glabrous skin become reduced following vincristine treatment since RA-type Aβ-LTMRs are mainly Meissner’s corpuscles in the glabrous skin of mice [31]. Piezo2 channels have been reported to be highly expressed in Meissner’s corpuscles to transduce tactile stimulation for the sense of touch in the glabrous skin [31, 32]. Piezo2-mediated MA currents in Merkel cells have been previously found to be suppressed in animals following vincristine treatment [33]. This raises the possibility that Piezo2-mediated mechanical transduction may also be suppressed in RA-type Nav1.8-negative Aβ-fiber mechanoreceptors, i.e., Meissner’s corpuscles, following vincristine treatment. Reducing mechanical sensitivity in Meissner’s corpuscles by vincristine treatment may be a cause of the numbness in vincristine-induced neuropathy.

SA-type Nav1.8-negative Aβ-fiber mechanoreceptors, which are mainly Aβ-LTMRs, are most likely to be Merkel cell-neurite complexes in the glabrous skin of mice [31]. Previous studies have shown that Piezo2 channels are mechanical transducers expressed on Merkel cells and their neurites [34–36]. We have previously demonstrated that vincristine treatment significantly reduces Piezo2-mediated MA currents in Merkel cells and reduces the SA-type impulses in whisker hair follicles [33]. However, in the present study with the hindpaw glabrous skin of mice, vincristine treatment did not affect the mechanical sensitivity of SA-type Nav1.8-negative Aβ-fiber mechanoreceptors. This discrepancy could be due to the difference in vincristine doses between the present study and our previous study [33].

Similar to Nav1.8-ChR2-negative Aβ-fiber mechanoreceptors, Nav1.8-ChR2-negative Aδ-fiber mechanoreceptors also predominantly LTMRs displayed either RA-type or SA-type responses, with RA-type being the majority type. A previous study has suggested that Aδ-LTMRs are D-hair mechanoreceptors in the glabrous skin of rodents [37]. Interestingly, vincristine treatment reduced the numbers of RA-type and increased the numbers of SA-type of Nav1.8-ChR2-negative Aδ-fiber mechanoreceptors. This change may be due to the conversion of RA-type to SA-type of Nav1.8-ChR2-negative Aδ-fiber mechanoreceptors. A similar change has been observed in Aβ-LTMRs in the glabrous skin of mice after tissue inflammation [27]. Vincristine treatment did not affect the conduction velocity and mechanical sensitivity of Nav1.8-ChR2-negative Aδ-fiber mechanoreceptors. This finding suggests that Nav1.8-ChR2-negative Aδ-fiber mechanoreceptors, i.e., Aδ-LTMRs, are less susceptible to the toxicity of vincristine at the dose used in the present study.

Different from Nav1.8-ChR2-negative Aβ- and Aδ-fiber mechanoreceptors, all Nav1.8-ChR2-positive Aβ- and Aδ-fiber mechanoreceptors show SA-type impulses, a result consistent with our previous study [25]. In the control group, most Nav1.8-ChR2-positive Aβ- and Aδ-fiber mechanoreceptors are HTMRs with von Frey threshold ≥ 4 mN, indicating that they are Aβ- and Aδ-fiber mechanonociceptors. However, some Nav1.8-ChR2-positive Aβ- and Aδ-fiber mechanoreceptors had von Frey threshold < 4 mN, and belong to LTMRs rather than HTMRs. We found that the von Frey threshold in Nav1.8-ChR2-positive Aβ-fiber mechanoreceptors was significantly reduced following vincristine treatment. This indicates mechanical hypersensitivity in nociceptive Aβ-mechanoreceptors following vincristine treatment. This may be an underlying mechanism of mechanical allodynia/hyperalgesia in vincristine-induced neuropathy.

All Nav1.8-ChR2-positive C-fiber mechanoreceptors display SA-type impulses in response to mechanical stimulation. We show that the conduction of these C-fiber mechanoreceptors was significant reduced following vincristine treatment. Nav1.8-ChR2-positive C-fiber mechanoreceptors consist of both C-LTMRs and C-HTMRs [25]. We observed no significant difference in the von Frey threshold between the control and vincristine treated groups. This lack of the effect of vincristine on the mechanical sensitivity could be due to a potential opposite impact of vincristine on the mechanical sensitivity of C-LTMRs and C-HTMRs. A future study using transgenic mice whose C-LTMRs and C-HTMRs are selectively tagged with ChR2 can help to determine whether mechanical sensitivity is reduced in C-LTMRs and increased in C-HTMRs.

The present study has also characterized opto-sensitivity of Nav1.8-ChR2-positive Aβ-, Aδ-, and C-fiber mechanoreceptors. Interestingly, light stimulation only evoked RA-type impulses in Nav1.8-ChR2-positive Aβ-fiber mechanoreceptors, both RA-type and SA-type impulses in Nav1.8-ChR2-positive Aδ-fiber mechanoreceptors, and primarily SA-type impulses in Nav1.8-ChR2-positive C-fiber mechanoreceptors. The impulse types in response to light stimulation in Nav1.8-ChR2-positive Aβ-, Aδ-, and C-fiber mechanoreceptors were not significantly affected by vincristine treatment. Opto-sensitivity was also not affected by vincristine treatment. These results may suggest that the nerve endings of Nav1.8-ChR2-positive Aβ-, Aδ-, and C-fiber mechanoreceptors remain intact following vincristine treatment in the present study. Therefore, the changes in the mechanical sensitivity of Nav1.8-ChR2-negative Aβ-fiber mechanoreceptors and Nav1.8-ChR2-positive Aβ-fiber mechanoreceptors are unlikely due to potential damage of the nerve endings by vincristine treatment.

In conclusion, the present study has revealed that vincristine treatment reduces mechanical sensitivity of RA-type Nav1.8-ChR2-negative Aβ-fiber mechanoreceptors, most likely Meissner’s corpuscle LTMRs. This provides a putative mechanism underlying the numbness in vincristine-induced neuropathy. The present study also suggests that vincristine treatment may enhance the mechanical sensitivity of Nav1.8-ChR2-positive Aβ-fiber mechanoreceptors, most likely Aβ-fiber mechanonociceptors or HTMRs. This may contribute to mechanical allodynia/hyperalgesia in vincristine-induced neuropathy. Further investigations will be needed to fully understand how vincristine reduces the mechanical sensitivity of RA-type Aβ-LTMRs and increases the mechanical sensitivity of Aβ-HTMRs.

## Data Availability

No datasets were generated or analysed during the current study.
